# Generation of Replication-Proficient Influenza Virus NS1 Point Mutants with Interferon-Hyperinducer Phenotype

**DOI:** 10.1371/journal.pone.0098668

**Published:** 2014-06-02

**Authors:** Maite Pérez-Cidoncha, Marian J. Killip, Víctor J. Asensio, Yolanda Fernández, José A. Bengoechea, Richard E. Randall, Juan Ortín

**Affiliations:** 1 Department of Molecular and Cellular Biology, Centro Nacional de Biotecnología (CSIC), Madrid, Spain; 2 School of Biology, Centre for Biomolecular Sciences, University of St Andrews, St Andrews, United Kingdom; 3 Fundació d'Investigació Sanitària de les Illes Balears (FISIB), Bunyola, Mallorca, Spain; 4 Laboratory Microbial Pathogenesis, Fundació d'Investigació Sanitària de les Illes Balears (FISIB), Bunyola, Mallorca, Spain; 5 Ciber de Enfermedades Respiratorias (ISCIII), Madrid, Spain; The Scripps Research Institute, United States of America

## Abstract

The NS1 protein of influenza A viruses is the dedicated viral interferon (IFN)-antagonist. Viruses lacking NS1 protein expression cannot multiply in normal cells but are viable in cells deficient in their ability to produce or respond to IFN. Here we report an unbiased mutagenesis approach to identify positions in the influenza A NS1 protein that modulate the IFN response upon infection. A random library of virus ribonucleoproteins containing *circa* 40 000 point mutants in NS1 were transferred to infectious virus and amplified in MDCK cells unable to respond to interferon. Viruses that activated the interferon (IFN) response were subsequently selected by their ability to induce expression of green-fluorescent protein (GFP) following infection of A549 cells bearing an IFN promoter-dependent GFP gene. Using this approach we isolated individual mutant viruses that replicate to high titers in IFN-compromised cells but, compared to wild type viruses, induced higher levels of IFN in IFN-competent cells and had a reduced capacity to counteract exogenous IFN. Most of these viruses contained not previously reported NS1 mutations within either the RNA-binding domain, the effector domain or the linker region between them. These results indicate that subtle alterations in NS1 can reduce its effectiveness as an IFN antagonist without affecting the intrinsic capacity of the virus to multiply. The general approach reported here may facilitate the generation of replication-proficient, IFN-inducing virus mutants, that potentially could be developed as attenuated vaccines against a variety of viruses.

## Introduction

The influenza A viruses are human pathogens causing annual epidemics and occasional pandemics of respiratory disease that have a large public health and economic impact [1], [2]. As members of the *Orthomyxoviridae* family they have a segmented, single-stranded RNA genome of negative polarity [3]. These RNA molecules are assembled into ribonucleoprotein particles (RNPs), containing the viral polymerase and multiple nucleoprotein (NP) monomers [4], [5], that are responsible for viral transcription and replication [6]–[8]. Interestingly, these processes take place in the nuclei of infected cells, which implies numerous interactions with the gene expression machinery of the cell [9]–[13]. In fact, viral transcription is dependent on RNA polymerase II activity [14], [15], consistent with the use of cell-derived capped oligonucleotides as primers for viral mRNA synthesis [16], [17]. Viral RNA replication generates progeny RNPs similar to those present in the virion [5]–[8] that are exported from the nucleus by a CRM1-dependent pathway [18], [19].

The process of virus infection is detected by the cell using sensors collectively named pattern recognition receptors -PRRs- [20] that recognize virus-specific patterns (pathogen-associated molecular patterns -PAMPs-). Among these sensors, RIG-I is relevant during influenza virus infection [21]–[23] and recognizes regions of dsRNA containing a 5′-triphosphate [24], [25]. Activated RIG-I signals downstream by interacting with the mitochondrial protein IPS-1/MAVS/VISA/Cardif, which leads to the activation of IRF3 and NF-kB, and subsequently the production of type I IFN (reviewed in [26]. Type I IFNs (IFN-α/β) are a group of cytokines that bind the IFN-α/β receptor, resulting in activation of the Jak/STAT pathway and subsequent transcription of many IFN-stimulated genes (ISGs). Some of these genes, like Mx, OAS and PKR, show direct antiviral activity and reduce the production of virus progeny [27], [28].

Most viruses have evolved countermeasures to limit or delay the cellular innate immunity by blocking the activation or signaling of PRRs, inhibiting IFN signaling from the IFN receptor or directly inhibiting the activity of one or several antiviral ISGs (reviewed in [28]. In addition, many viruses down-regulate transcription and/or translation of cellular genes and hence indirectly inhibit the induction of IFNs and ISGs. In the case of influenza A viruses, the anti-IFN response is polygenic as mutations in many viral genes diminish viral replication efficiency in IFN responsive cells [29] and it has been reported that alterations in the polymerase or PB1-F2 genes appear to modulate the response [30], [31]. However, the multifunctional and non-structural NS1 protein is the principal IFN-counteracting factor of influenza viruses; thus, viruses lacking NS1 protein expression can not multiply in normal cells but are viable in cellular systems deficient in innate immunity (although viral yields are reduced) [32], [33]. NS1 can bind many proteins and RNAs, and although it is non-essential for virus replication, it has roles in viral protein synthesis, viral RNA replication, virion production and can modulate cellular post-transcriptional RNA processing and transport [34]–[38] (reviewed in [39]. The anti-IFN action of NS1 is exerted through a combination of several possible NS1-host cell interactions, such as: (i) down-regulation of new cellular transcription elongation and post-transcriptional RNA processing after infection [40]–[42], (ii) inhibition of RIG-I activation [21]–[23], [43], (iii) interference with the IFN signaling [44], [45] and (iv) direct inhibition of specific ISGs, like PKR and RNAse L [46]–[48].

Here we report a random mutagenesis approach to identify positions in the influenza A NS1 protein that can modulate the IFN response upon infection. Individual mutant viruses were selected using a reporter cell-line in which GFP expression was under the control of the IFN-β promoter. Isolated viruses led to increased levels of IFN expression and a reduced capacity to counteract exogenous IFN during infection. Sequence analysis revealed that these viruses contained mutations throughout the NS1 protein.

## Materials and Methods

### Biological materials

The influenza A strains A/Victoria/3/75 (VIC) and ΔNS1 [32] were used throughout. Encephalo-myocarditis virus (EMCV) was used for IFN bioassay. The MDCK cell line was purchased from the ATCC and the A549 cell line [49] was obtained from J.A. Melero. The generation of MDCK-V2 and A549/pr(IFN-β).GFP cells [50]–[52] has been described. The A549/pr(ISRE).Luc cells, stably expressing luciferase under an ISRE promoter were obtained from G. Adolf, Boehringer Ingelheim, Austria. They were further engineered to express BVDV/NPro (A549/pr(ISRE).Luc-BVDV-N^pro^) to render them IRF3-deficient and unable to generate IFN [53]. Antibodies used included monoclonal antibodies specific for β-actin (Sigma) and phospho-Akt (Ser473; Cell Signaling Technology) as well as polyclonal antibodies to ISG56 (Santa Cruz), MxA (Santa Cruz), phospho-IRF3 (Ser396; Cell Signaling Technology), Akt (pan; Cell Signaling Technology), cleaved Caspase-3 (Asp175; Cell Signaling Technology) and Stat1 (Cell Signaling Technology). The specific anti-NS1 and anti-NP rabbit antibodies [54], [55], anti-M1 mouse antibodies [56], anti-NS1 rat antibodies [35] and anti-V2 antibodies [51] have been described previously.

### Virological techniques

Influenza virus plaque assay was carried out on MDCK cells as described [57]. Viral plaques were revealed either by staining with crystal violet or by immunocytochemistry using sheep anti-influenza sera (anti-X31; Diagnostics Scotland) [58]. The antiviral activity of culture supernatants was determined by cytopathic effect (CPE)-reduction bioassay. Culture supernatants from cells infected at 5 PFU/cell were harvested at 24 hpi and centrifuged at 1500×g for 10 min to eliminate cellular debris. After UV-treatment to inactivate residual virus, the supernatants were serially diluted 2-fold and added to A549/BVDV-Npro cell monolayers for 24 h prior to infection with ECMV at 0,05 PFU/cell. The monolayers were fixed 2–3 days thereafter and CPE was determined by crystal violet staining. The number of wells protected from infection were converted to IFN bioassay units using an IFN-α standard.

### Generation of a mutant virus library

A NS segment mutant library was generated by mutagenic PCR using 19.4 µM dPTP and 1.5 µM 8-oxo-dGTP. Amplification was performed for 10 cycles comprising 40″/55°C and 1 min/72°C followed by a final step of 7 min/72°C using as primers the terminal sequences of NS segment (5′-AGCAAAAGCAGGGTGACAAA-3′ and 5′- ACAAGGGTGTTTTTTATCAT-3′). The mutated PCR product was used as template for a 30-cycles PCR reaction in the same conditions but without mutagenic analogues, in which the primers contained BsmB1 terminal sequences (5′- GTCACGTCTCATATTAGTAGAAACAAGGGTGTTTTTTATC-3′ and 5′- CTGACGTCTCAGGGAGCAAAAGCAGGGTGACAAAGAC-3′). The final PCR product was cloned into the BsmB1 sites of pHH21 vector [59] and the plasmid library was amplified by high-density colony plating. The plasmid mutant library was used to generate recombinant NS RNPs by co-transfection of HEK293T cells with plasmids expressing viral NP, PB1, PA and his-tagged PB2 [60]. The recombinant RNP library was purified from the transfected cells by Ni^2+^-NTA agarose affinity chromatography as described earlier [60]. To rescue the recombinant RNPs into infectious virus, cultures of HEK293T cells were first transfected with a mixture of plasmids expressing PB1, PB2, PA and NP [60]. At 24 hours post-transfection, the cells were further transfected with a mixture of virion RNPs purified by glycerol density centrifugation [61] and a molar excess of purified recombinant NS RNPs, using cationic liposomes [62]. Finally, at 16 hours post-transfection of RNPs the cells were trypsinised, mixed with a 10-fold excess of MDCK-V2 cells and plated. When the mixed culture formed a monolayer, trypsin was added to final concentration of 2.5 µg/ml and the cells were incubated until complete cytopathic effect was apparent.

### Mutant virus screening

For cell sorting, A549/pr(IFN-β).GFP cells were infected at 0.04 FPU/cell. At 8 hpi, cells were trypsinised, resuspended in Mg^2+^- and Ca^2+^-free PBS and passed through a 30 µm pore filter to obtain single-cell suspensions. Cell sorting was carried out using an Influx cell sorter (BD Bioscience) equipped with 5 solid-state lasers. eGFP was excited with 488 nm laser and collected through a 517/30 filter. A Forward Scatter versus Side Scatter plot was used for excluding debris. Data from list files of 10.000 events were analyzed using Summit 4.3 Software (DAKO). FACS analysis was performed with A549/pr(IFN-β).GFP cells trypsinised to obtain single-cell suspension and fixed in PBS/1% formaldehyde. GFP expression was examined using a BD FACScan flow cytometer and data were analyzed using FlowJo (Treestar).

### Protein analyses

Western blotting and immunofluorescence were carried out as described previously [63], [64]. For immunofluorescence, the cultures were washed with PBS, fixed with 1% paraformaldehyde and permeabilized with 0.5% Triton X100 for 5 min. The cells were blocked with PBS-3%BSA and incubated with primary antibody diluted in PBS-0.1% BSA, for 1 h at room temperature. After washing with PBS, the preparations were further incubated with goat anti-rabbit, or goat anti-rat antibodies bound to Alexa 488 or Alexa 594 fluorochromes. The preparations were mounted in Prolong reagent and analyzed by confocal microscopy using a Leica TSC SP5 microscope. For luciferase assay A549/pr(ISRE).Luc or A549/pr(ISRE).Luc-BVDV-N^pro^ were infected at 5 FPU/cell and 7 hours later were treated or not with IFN-α (Roferon A, Roche) at 10^4^ units/ml. At 13 hours post-infection luciferase expression was determined using the Luciferase Assay System (Promega).

### RNA analyses

Virion RNA was purified by treatment with 0.5% SDS and 200 µg/ml proteinase K in TNE for 30 min at 37°C followed by extraction with phenol-chloroform-isoamylalcohol-hydroxyquinolein and ethanol precipitation [65]. Purified RNA was used for RT-PCR using segment-specific terminal oligonucleotides and the Titan One PCR system. The reverse transcription reaction was performed for 30 min at 42°C and then the PCR was performed for 30 rounds of 94°C for 30 s, 55°C for 30 s, and 68°C for 2′30 s with a final extension time of 7 min at 68°C.

## Results and Discussion

### Generation and screening of a library of virus mutants affected in the NS RNA segment

**Figure 1:**
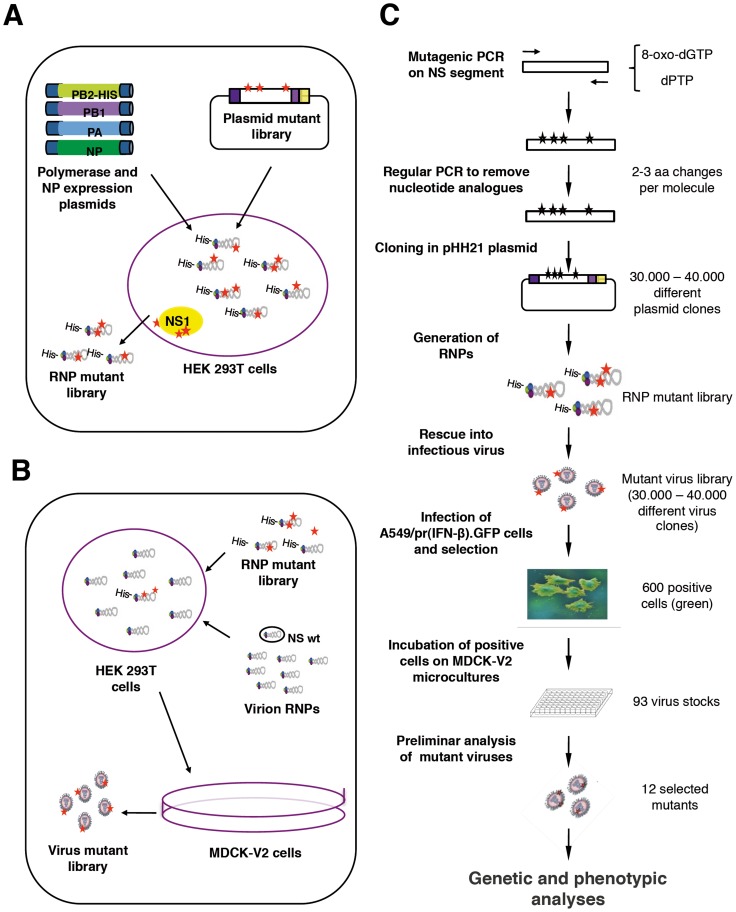
Diagram of the generation of a library of virus mutants affected in the NS RNA segment. (A) A NS cDNA segment was mutagenised by PCR and cloned into the pHH21 genomic vector [59]. Recombinant NS RNPs were obtained by co-transfection of the plasmid library with plasmids expressing the polymerase subunits and the NP into HEK293T cells. (B) The recombinant NS mutant RNPs were purified by Ni-NTA-agarose chromatography and co-transfected into HEK293T cells together with purified wt virion RNPs. The transfected HEK293T cells were then co-cultured with an excess of MDCK-V2 permissive cells until cytopathic effect was apparent. (C) Diagram of the steps carried out for the generation of virus mutant library and the subsequent virus selection.

**Figure 2:**
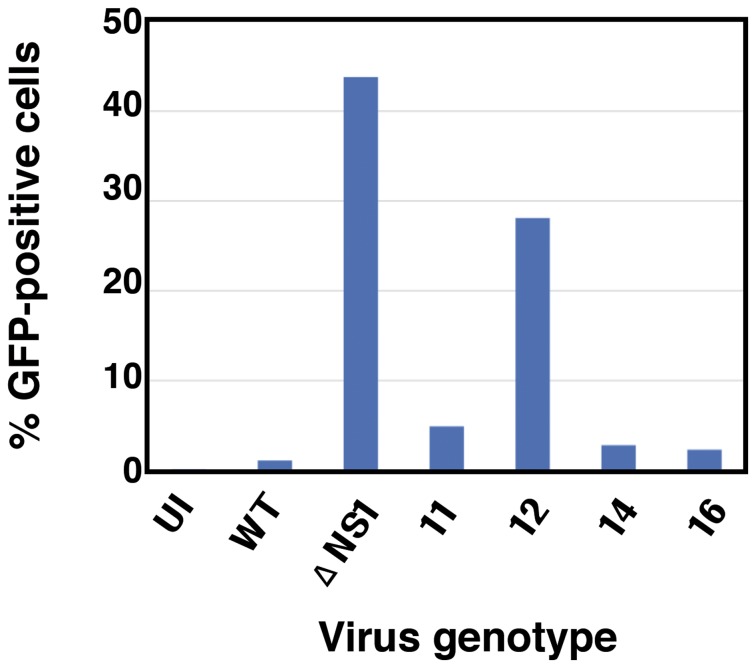
Induction of IFN-dependent GFP by infection with virus mutants. Cultures of A549/pr(IFN-β).GFP cells were infected at 5 FPU/cell and at 8 hpi they were trypsinised to obtain single-cell suspension, fixed in PBS/1% formaldehyde and subjected to FACS analysis as indicated in Materials and Methods.

As the NS1 protein is the main countermeasure used by influenza A viruses to overcome cellular innate immune responses (reviewed in [26], [39], we used it as a target to generate mutants potentially compromised with regards to the virus interplay with these cellular responses. The experimental strategy involved random mutagenesis, rescue of replication-proficient mutants in cells deficient in the IFN response and subsequent screening for viruses with an enhanced capacity to induce IFN upon infection. We first generated a library of mutants affected in the NS segment, that encode NS1 and NS2, by mutagenic PCR. cDNA of influenza A/Victoria/3/75 (H3N2) (VIC) virus segment 8 was PCR amplified using the appropriate concentrations of pPTP and 8-oxo-dGTP to obtain an average of 4 nucleotide changes per molecule, as determined by sequencing random plasmid clones obtained after cloning the PCR product in the pHH21 vector [59]. A NS plasmid library containing approximately 40.000 independent clones was amplified by high-density plating and used to generate a library of recombinant NS RNPs by replication in HEK293T cells expressing a His-PB2-tagged polymerase complex and NP (Fig. 1A). The mutants present in this RNP library were transferred to infectious virus by RNP competition as described [35], i.e. cultures of HEK293T cells were co-transfected with purified VIC virion RNPs and an excess of the His-purified recombinant NS RNP mutant library. The co-transfected cells were then plated onto MDCK-V2 cells to allow virus amplification (Fig. 1B). These cells express the V protein of parainfluenza virus type 2 (PIV2) and are insensitive to IFNα/β, since STAT1 is degraded and IFN signaling is blocked (Supplementary Fig. S1) [52]. Under the conditions used neither the generation of the RNP library nor its transfer to infectious virus would restrict the genetic complexity of the original plasmid library and control experiments showed that around 70% of the virus rescued contained a mutant instead of the wt VIC NS segment (data not shown). To identify potential virus mutants affected in viral IFN antagonism, the virus mutant library was used to infect A549 cells engineered to express GFP under control of IFN promoter (A549/pr(IFN-β).GFP cells) [50] at low multiplicity of infection, using wt virus as a control. Under these conditions the possibility of cell coinfection by two mutants or by potential defective-interfering (DI) particles present in the sample was diminished. The infected cell population was sorted for GFP expression and individual positive cells were plated on microcultures of MDCK-V2 cells. From around 600 positive cells plated, 93 gave rise to a normal infection in MDCK-V2 cells. These were subjected to preliminary characterization by flow-cytometry after high-multiplicity infection of A549/pr(IFN-β).GFP cells, by determining the relative virus yield in MDCK-V2 *versus* MDCK cells and testing for ISG induction. As a result 12 virus mutants were chosen for sequencing, seven of which contained mutations in the NS segment and 5 did not. This observation is not surprising, as a small amount of wt virus-infected cells led to GFP expression above background levels (see Fig. 2 below), and was interpreted as the result of spontaneous IFN-inducing mutations present in the virus population. Finally, 4 virus mutants (ns 11, 12, 14 and 16) were considered most interesting and were selected for further analyses. The induction of GFP observed after high-multiplicity infection is presented in Fig. 2. Particularly relevant were the results obtained after infection with mutant 12, that produced a proportion of GFP-positive cells almost as high as that induced by infection with ΔNS1 virus mutant [32], used as a control.

### Genotypic and phenotypic analyses of the selected virus mutants

The sequence analysis of the virus clones containing alterations in the NS RNA segment was consistent with the mutagenesis used, i.e. each mutant contained 1 to 5 nucleotide changes, some of which led to amino acid changes. Among these, 9 mutations were detected in NS1 and 5 in NEP(NS2) protein. All but one of the mutations affecting NS1 protein were mapped to positions very conserved phylogenetically (Table 1), suggesting that these could be relevant for the mutant phenotype.

**Table 1:** Mutations detected in individual virus mutants.

MUTANT	POSITION	NT CHANGE	AA CHANGE NS1	CONSERVATION	AA CHANGE NS2	CONSERVATION
**1**	**551<\emph>**	**A-G**	**-**	**-**	**K18S**	**1N/4828**
	**584**	**A-G**	**-**	**-**	**N29S**	**4S/4828**
**7**	**408**	**A-G**	**I128V**	**3A/6667**	**n/a**	**-**
	**740**	**A-G**	**STOP238W**	**0W/6667**	**E81G**	**1D/4828**
	**780**	**T-C**	**n/a**	**-**	**-**	**-**
**10**	**844**	**T-C**	**-**	**-**	**F116L**	**5L/4828**
**11**	**393**	**A-G**	**I123V**	**V**	**n/a**	**n/a**
	**496**	**T-C**	**V157A**	**3/6667**	**n/a**	**n/a**
	**609**	**T-C**	**S195P**	**0/6667**	**-**	**-**
**12**	**152**	**C-A**	**-**	**-**	**n/a**	**n/a**
	**540**	**G-A**	**E172K**	**7/6667**	**-**	**-**
**14**	**94**	**T-C**	**V23A**	**V**	**n/a**	**n/a**
**16**	**229**	**T-C**	**I68T**	**0/6667**	**n/a**	**n/a**
	**261**	**A-G**	**M79V**	**0/6667**	**n/a**	**n/a**
	**392**	**A-G**	**-**	**-**	**n/a**	**n/a**
	**476**	**C-T**	**-**	**-**	**n/a**	**n/a**
	**557**	**A-G**	**-**	**-**	**Q20R**	**22/4828**

The mutations detected in individual virus mutants are presented. The nucleotide changes observed in the NS segment are indicated, as well as the corresponding amino acid substitution in the NS1 or NEP ORFs. The conservation of these positions in the influenza sequence database is presented as the number of instances that the mutant amino acid observed appears among the total number of sequences screened.

**Figure 3:**
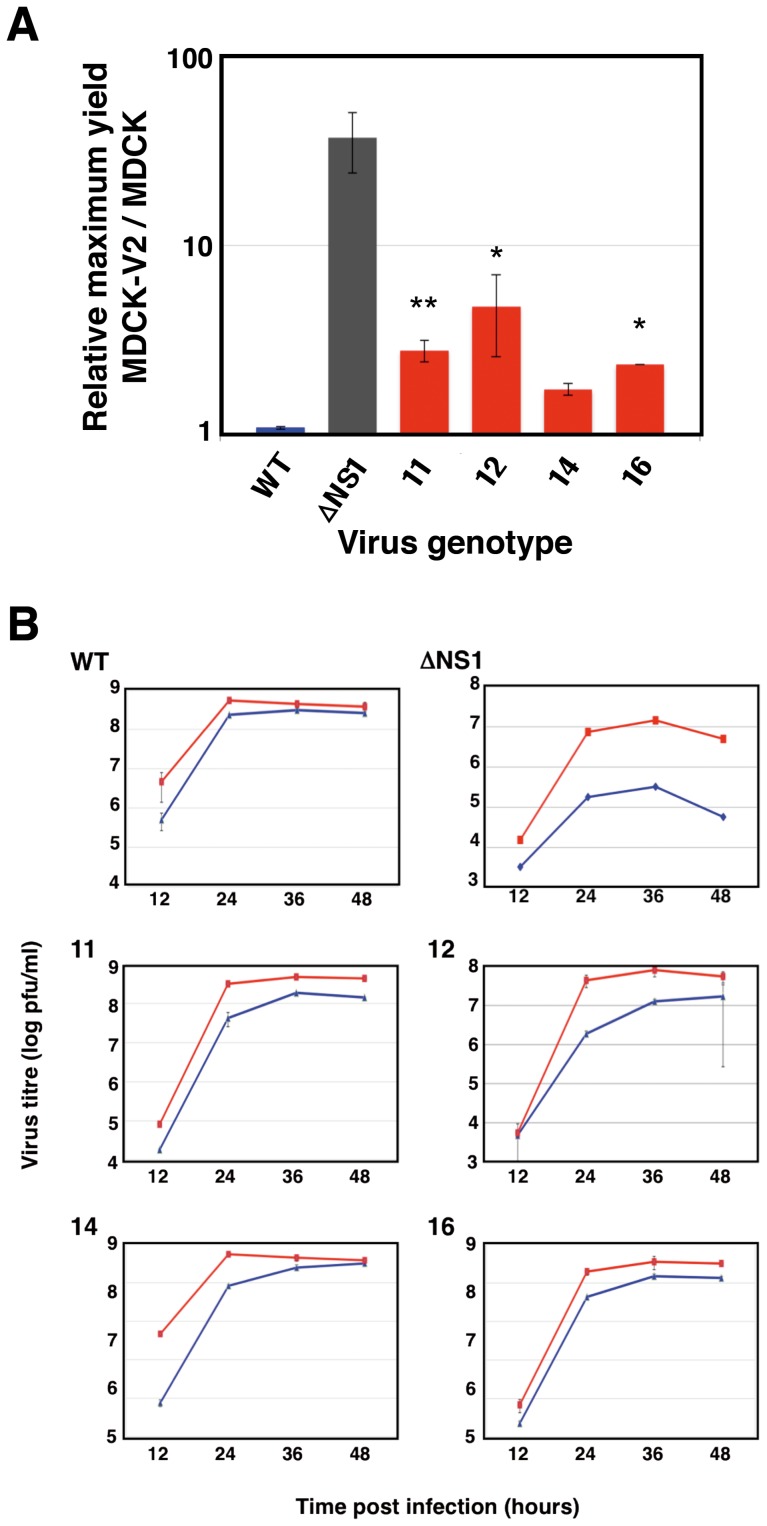
Efficiency of virus replication in IFN-responsive and IFN non-responsive cells. Cultures of either MDCK or MDCK-V2 cells were infected with the various viruses at a moi of 0.001 pfu/cell. At various times after infection samples of the supernatant media were withdrawn and virus titer was determined in MDCK-V2 cells. (A) The ratio of maximal titers obtained from MDCK-V2 versus MDCK infected cells is presented. The values are averages and standard deviations of 3 independent experiments. (B) Kinetics of virus growth in MDCK-V2 (red lines) or MDCK (blue lines) as determined by plaque-assay in MDCK-V2 cells. The titers are averages and standard deviations of 3 independent experiments. * p-value<0.05. ** p-value<0.01.

First, the replication efficiency of the selected mutant viruses in cells proficient or deficient in IFN response was ascertained by determination of the kinetics of virus multiplication in low-multiplicity infections. The relative maximal yield in MDCK-V2 versus MDCK cells for several experiments are shown in Fig. 3A. All mutants produced higher titers in IFN-deficient cells and were fully competent for replication in MDCK cells, although mutant 14 showed a protracted kinetics (Fig. 3B). To analyze to what extent these mutations modified the virus infectious cycle in IFN competent cells, the accumulation and localization of NS1 protein were determined. No major alterations in NS1 protein localization were observed, although mutant 12 NS1 protein accumulated to slightly reduced levels (Fig. S2). These results are consistent with the normal replication kinetics and high titers obtained with most of these mutant viruses (Fig. 3B) and confirm that they show good replication fitness.

### Interaction of the NS1 virus mutants with the host innate immune system

**Figure 4:**
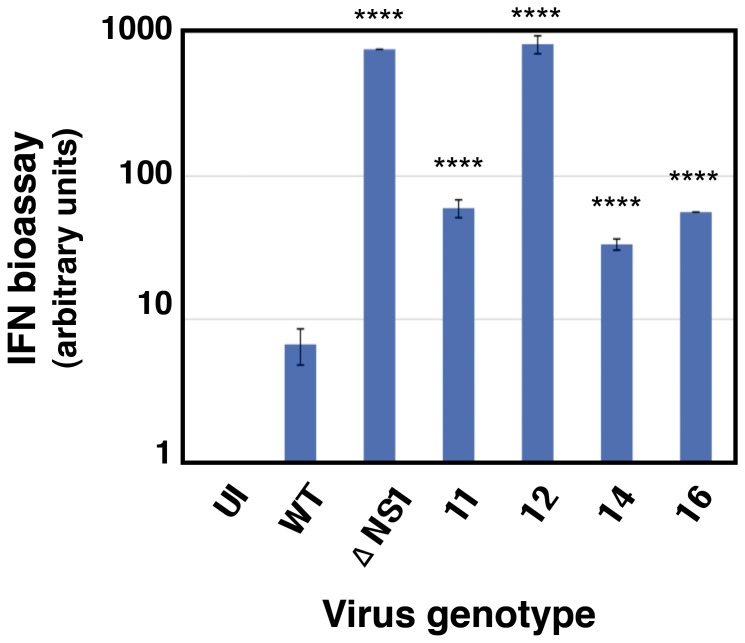
Secretion of antiviral factors by wt and mutant virus infection. Cultures of A549 cells were infected at a moi of 5/cell and at 24 hpi the culture supernatants were collected and their activity to interfere with ECMV infection of A549/BVDV-Npro cells was determined by end-point dilution, using purified alpha-IFN as standard. **** p-value<0.0001.

**Figure 5:**
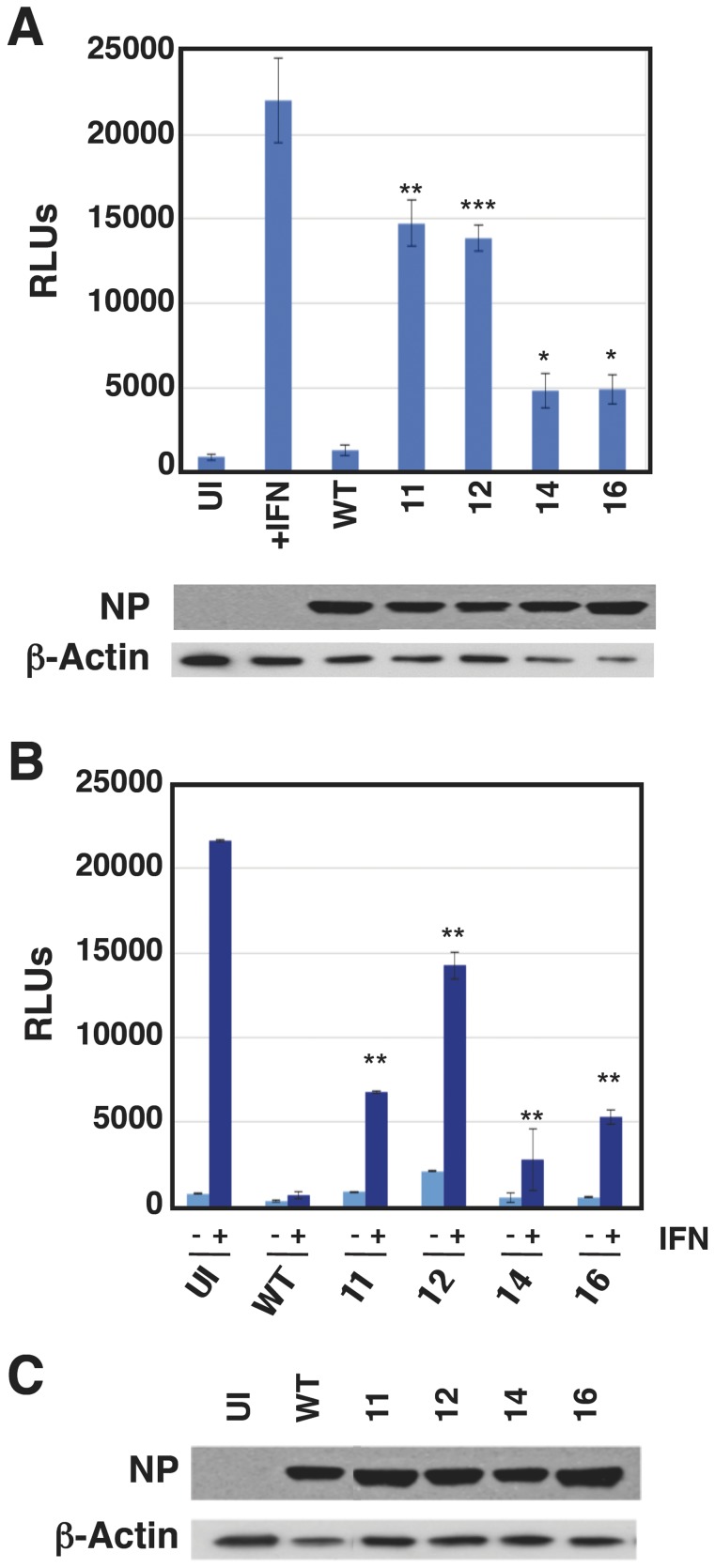
Induction of IFN-dependent luciferase by wt and mutant viruses. Cultures of A549/pr(ISRE).Luc (A) or A549/pr(ISRE).Luc-Npro cells (B) were infected at a moi of 5 pfu/cell with the mutants indicated, wt virus as reference or mock infected. At 7 hpi IFN was added to the indicated samples and at 13 hpi total cell extracts were prepared and the luciferase activity was determined as indicated in Materials and Methods. The progress of influenza infection was verified by determination of NP accumulation, using β-actin as loading control. * p-value<0.05. ** p-value<0.01. *** p-value<0.001.

The screening method used to identify the viral mutants, and the induction of GFP after infection with those selected for analysis (Fig. 2), suggested that they induced a strong IFN response. To directly test this prediction, the interfering IFN activity in the supernatants of cells infected with the mutants was compared to the activity in supernatants from cells infected with wt or ΔNS1 viruses. The results are presented in Fig. 4 and indicate that all mutants induced at least ten-fold higher levels of IFN than wt virus. Consistent with the GFP expression data, mutant 12 led to an antiviral activity similar to that obtained with ΔNS1 virus. Next, we tested the downstream consequences of the antiviral activity observed after infection with the mutant viruses. To assay the induction of IFN-stimulated gene (ISG) expression, cultures of A549 ISRE-Luc cells, that express luciferase under control of an IFN-inducible promoter, were infected with wt or each of the mutants and the amount of luciferase activity was determined. No increase in luciferase expression above background levels could be detected in wt virus-infected cells, yet all virus mutants induced luciferase in excess over wt virus, particularly mutants 11 and 12 (Fig. 5A). These results, together with those presented in Fig. 4, suggest that the mutants 11 and 12 are not only able to induce IFN but also are unable to block signaling downstream of the IFN receptor. To verify this hypothesis we used A549 NPro/ISRE-luc cells, which also express IFN-inducible luciferase but cannot produce IFN due to the expression of BVDV NPro protein that targets the IRF3 transcription factor for degradation; as such, none of the viruses used was able to induce luciferase expression in these cells (Fig. 5B). However, when exogenous IFN was added to the medium, large amounts of luciferase were expressed in the absence of infection and this induction was effectively blocked by wt virus infection. As predicted by the results shown above, mutants 11 or 12 were unable to block luciferase induction by IFN while mutants 14 or 16 did so partially, but much less efficiently than wt virus.

**Figure 6:**
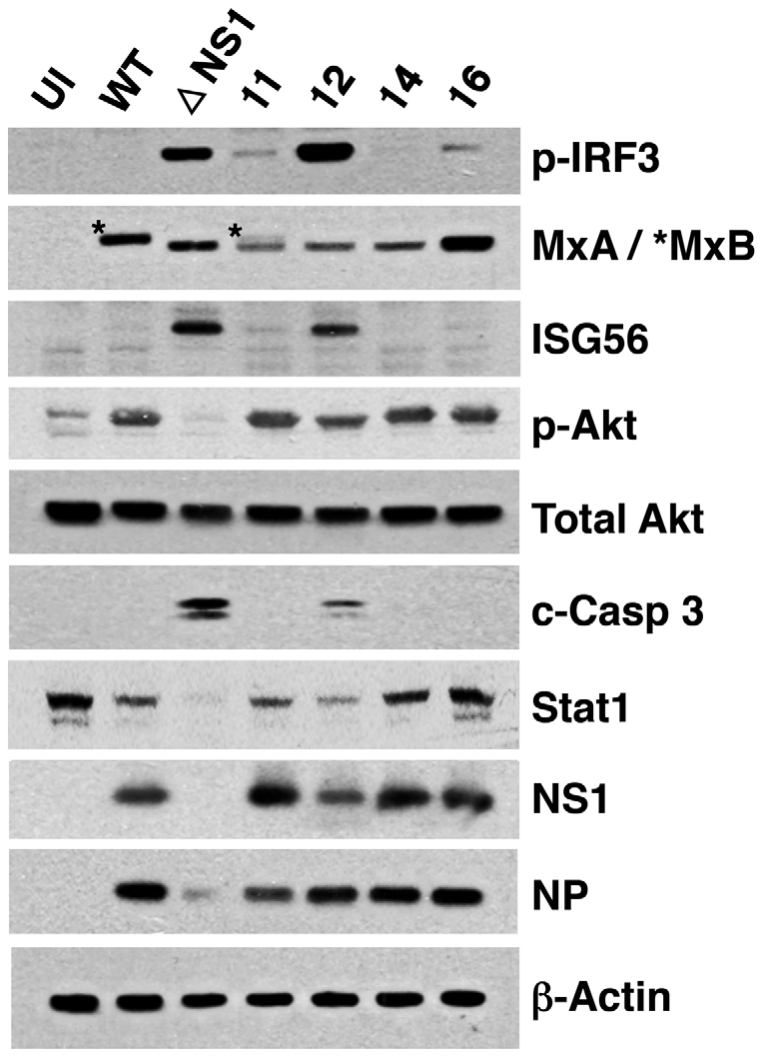
Cell-signaling activation by the infection of wt or mutant influenza viruses. The induction of ISGs (MxA, ISG56) and the activation of cell signaling (p-IRF3, p-Akt, c-Casp3, STAT1 and p-eIF2alpha) was analyzed by WB with specific antibodies in cells infected with the mutant viruses indicated, using uninfected cells (UI) and cells infected with wt or ΔNS1 viruses as references. The progression of virus infection was controlled by WB with antibodies specific for NS1 and NP, using β-actin as loading control.

To get a deeper understanding of the mutant phenotype in regard to the IFN response, a number of critical elements for IFN expression and signaling were further analyzed. As presented in Fig. 6, all mutants induced the phosphorylation of IRF3 to varying degrees. Mutant 12 did so as strongly as ΔNS1 virus, consistent with the IFN expression data presented in Fig. 4. Both ΔNS1 and mutant 12 viruses additionally induced high expression of ISG56/IFIT1, which is induced by activated IRF3 in an IFN-independent manner, as well as directly by IFN [66]. ΔNS1 and all mutants, but not wt virus, induced the expression of MxA, in agreement with the luciferase expression described in Fig. 5A. In addition, wt virus (and partly mutant 11) induced the expression of MxB protein, but this phenomenon was not analyzed further. We observed AKT phosphorylation with all our virus mutants, indicating that the ability of these NS1 mutants to bind and activate PI3K [67] was retained, unlike the situation in ΔNS1-infected cells where activation of the Akt pathway is eliminated due to lack of NS1 expression [67]–[69]. Furthermore, among the point mutants studied, only mutant 12 lead to a slight increase in apoptosis as indicated by caspase-3 cleavage.

**Figure 7:**
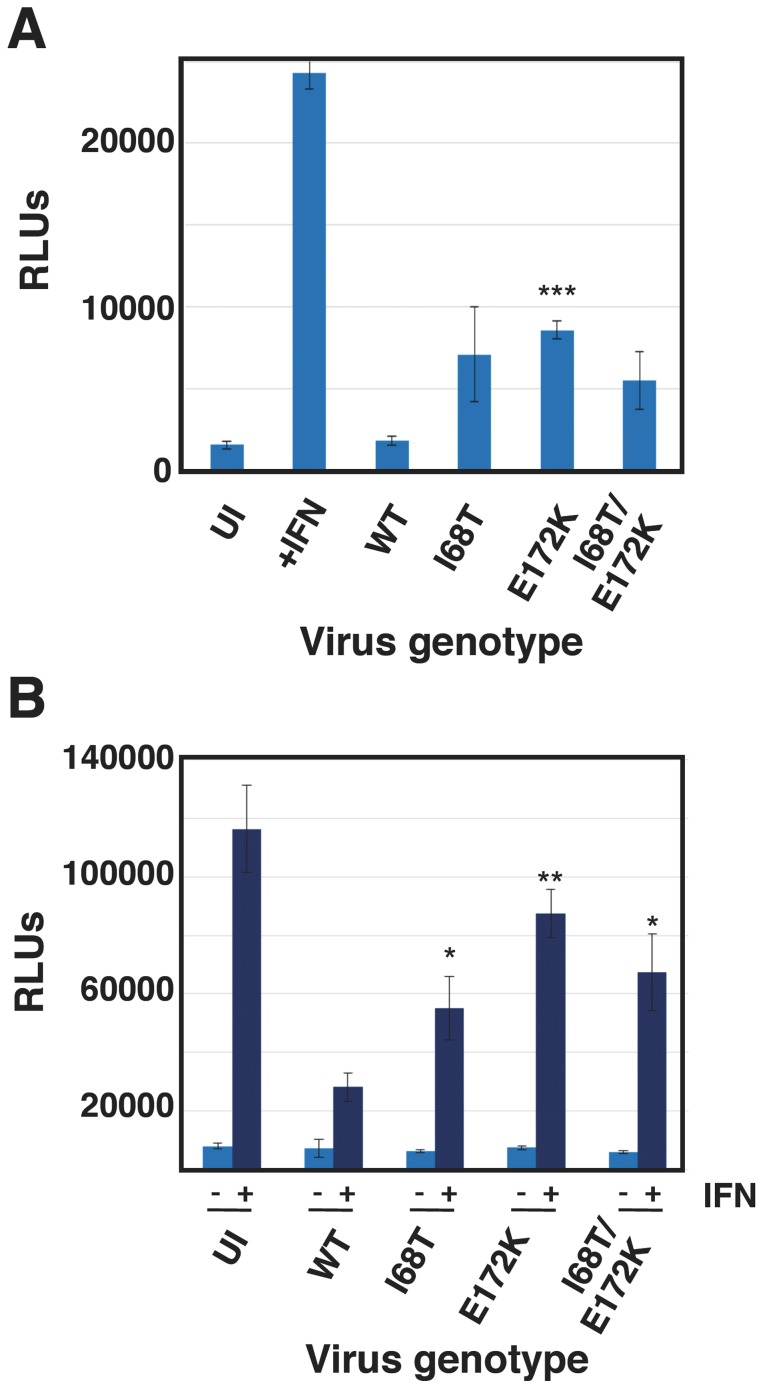
Induction of IFN-dependent luciferase by wt and recombinant viruses. Cultures of A549/pr(ISRE).Luc (A) or A549/pr(ISRE).Luc-Npro cells (B) were infected at a moi of 5 pfu/cell with the rescued recombinant mutants indicated, wt virus as reference or mock infected. At 7 hpi IFN was added to the indicated samples and at 13 hpi total cell extracts were prepared and the luciferase activity was determined as indicated in Materials and Methods. * p-value<0.05. ** p-value<0.01. *** p-value<0.001.

In order to verify whether the mutations identified in the NS1 mutants were relevant for the observed phenotype, some of them were rescued in infectious virus, using the VIC virus genetic background. Specifically, rescue of mutations NS1-I68T, NS1-E172K and NS1-I68T/E172K was attempted and all rescued viruses were viable. The expression of luciferase under control of an ISRE promoter was used to analyze the phenotype of these recombinant viruses and the results are presented in Fig. 7A. All three recombinant virus mutants showed luciferase expression above those observed for wt virus and, furthermore, they were less efficient than wt virus at counteracting the luciferase expression upon addition of exogenous IFN (Fig. 7B).

### The NS1 protein: A versatile blocker of the innate immune response

**Figure 8:**
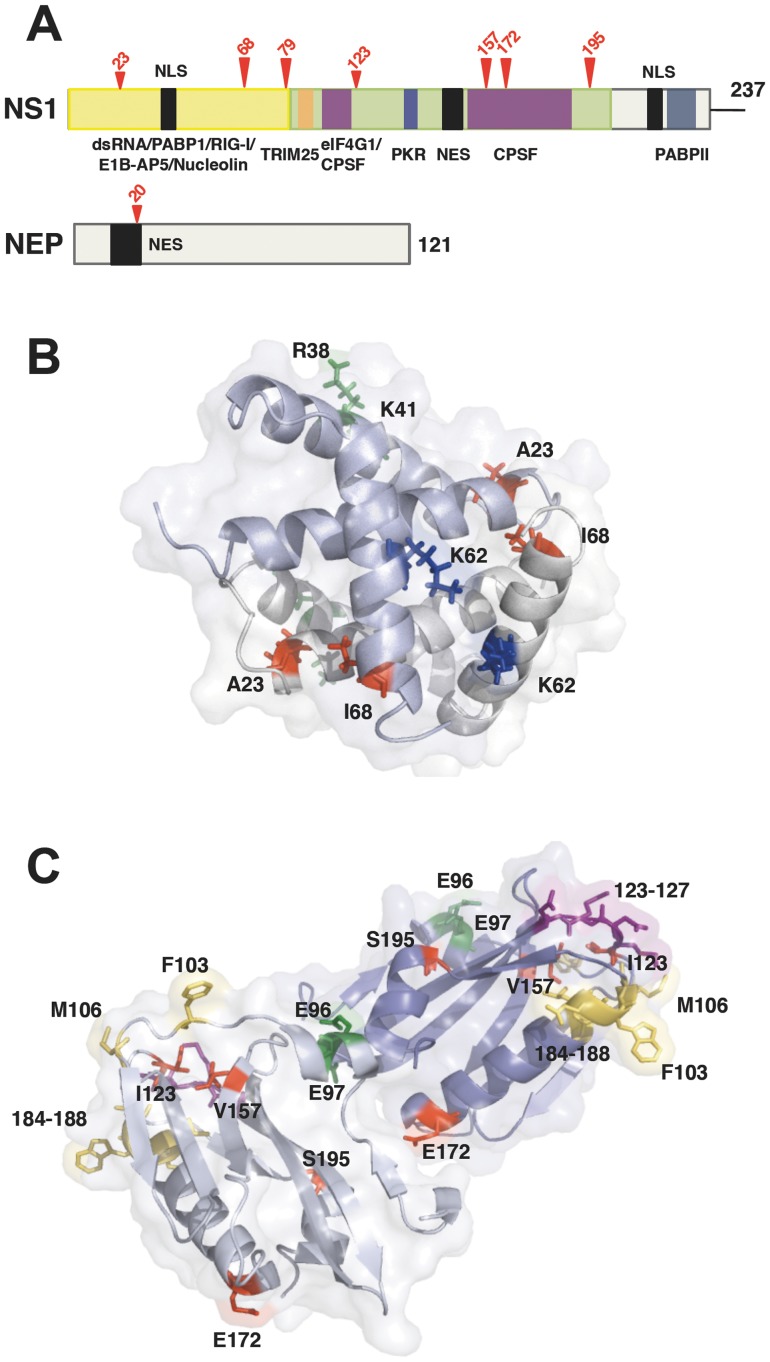
Localization of the NS1 mutations affecting the counteraction of the innate immune response to influenza infection. (A) Diagram showing the NS1 coding region including the position of the NLSs, the NES and the binding regions of NS1 interaction partners (dsRNA, PABP1, RIG-I, E1B-AP5, Nucleolin, TRIM25, eIFG1, PKR, CPSF and PABP2). The positions of the mutations identified in this report are indicated with red arrows, which are long for the positions evolutionary conserved among influenza viruses and short for those variable. The NEP coding region is also presented, including a mutation reported. (B) Atomic structure of the RNA-binding domain NS1 dimer showing the amino acids responsible for RNA binding [77], [78](R38 and K41, green), the position mutated in a temperature-sensitive mutant defective in PKR block [46](K62, blue), as well as those identified in this study as present in IFN-inducing viruses (A23, I68, red). (C) Atomic structure of the effector domain NS1 dimer showing the amino acids responsible for TRIM25 binding [43] (E96, E97, green), PKR binding [47], [74](123–127, purple), CPSF binding [72], [73], [75], [76](103, 106, 184–188, yellow) and those identified in this study as present in IFN-inducing viruses (I123, V157, E172 and S195, red).

The influenza NS1 protein is the main virus factor counteracting the cellular innate immune response [26], [32], [39], [40], [47]. NS1 exerts this function by one or several mechanisms, depending on the particular virus strain considered [70], including inhibition of RIG-I activation [21]–[23], [43], down-regulation of new cellular gene expression after infection [40], [71], blocking IFN signaling [44] or avoiding the activation of some ISGs, like PKR [47] or 2′-5′ OAS [48]. In this report we took an unbiased genetic strategy to define features in the NS1 gene that may be relevant for blocking the IFN induction or for counteracting IFN activity. The experimental approach included the generation of a large library of NS1 mutant viruses that are proficient for replication in IFN non-responsive cells and subsequent screening for those able to induce IFN-dependent GFP expression (Fig. 1). Using this approach, mutations that had deleterious affects on virus replication would be selected against. Indeed, although the selected mutant viruses replicated more efficiently in IFN-non-responsive cells than in their normal counterparts they still replicated to relatively high titers in MDCK cells (Fig. 3B). As expected, the mutant viruses induced higher levels of IFN and ISGs (Figs. 4, 5A and 6), and were compromised in their capacity to block the effects of added IFN (Fig. 5B). In previous work a number of mutations in NS1 have been described that affect interactions with dsRNA, RIG-I, PKR, TRIM25 or CPSF and reduce the capacity of the protein to counteract the innate immune response [43], [46], [47], [72]–[78]. These mutations localize to the RNA-binding domain or the effector domain and are depicted in Fig. 8B, C, as well as in the flexible linker sequence between them. In this study we used the VIC strain as parental virus that contains the conserved amino acid residues at all positions known to be involved in NS1 mediated IFN antagonism. The mutations obtained in this study did not affect these positions but, rather, they were localized elsewhere along the RNA-binding, linker and effector regions of the protein (Fig. 8A). The only exception was mutation I123V that affects one of the positions described in the PKR-binding region and alters the kinetics of viral RNA synthesis [47], [74]. Most of the mutations detected alter positions in NS1, which are very conserved in the sequence database (Fig. 8A, long arrows; Table 1), suggesting that they are relevant for the observed phenotype. Consistent with this, the introduction of these mutations into wt virus by reverse genetics led to enhanced ISG expression and defects in IFN counteraction similar to those observed in the original mutant viruses (Fig. 7).

All together, the results presented in this report indicate that, in the genetic background of the VIC strain, the NS1 protein as a whole is important for counteracting the innate immune response. Thus, mutations at either the RNA-binding domain (I68T) or the effector domain (E172K) diminish the capacity of the protein to perform optimally against IFN action. These results also constitute proof-of-concept showing that the approach described can yield mutant viruses that are replication-proficient in IFN non-responsive cells, but in IFN-competent cells induce high levels of IFN and fail to block the induction of ISGs by IFN. Furthermore, the general approach of using reporter cell-lines to select for viruses that are deficient in their ability to circumvent the IFN response but which are still replication competent may be applicable to other virus systems, thereby facilitating the isolation of attenuated vaccine candidates against a variety of viruses.

## Supporting Information

Figure S1 **Characterization of MDCK-V2 cells.** (A) Cultures of either MDCK-V2 (red lines) of MDCK (blue lines) cells were infected with wt (full line) or DNS1 (dotted line) virus at 0.001 pfu/cell. At the times indicated, samples of the supernatants were withdrawn and virus titres were determined in MDCK-V2 cells. (B) Extracts from MDCK or MDCK-V2 cells were treated or not with dog IFN and analysed by Western blot using anti-STAT1, anti-MxA, anti-V2 or anti-actin antibodies, as indicated.
(TIF)

Figure S2 **Intracellular localization of NS1 in wt- or mutant virus-infected cells.** Cultures of A549 cells were infected with wt or mutant viruses at 5 pfu/cell. At the times indicated the cultures were fixed and processed for immunofluorescence using antibodies specific for NS1. The figure shows projections of representative fields.
(TIF)
