# Supplementary figures and images for: Generation of Replication-Proficient Influenza Virus NS1 Point Mutants with Interferon-Hyperinducer Phenotype
Source: PLoS One. 2014 Jun 2;9(6):e98668. doi: 10.1371/journal.pone.0098668 (PMC4041880; doi:10.1371/journal.pone.0098668)

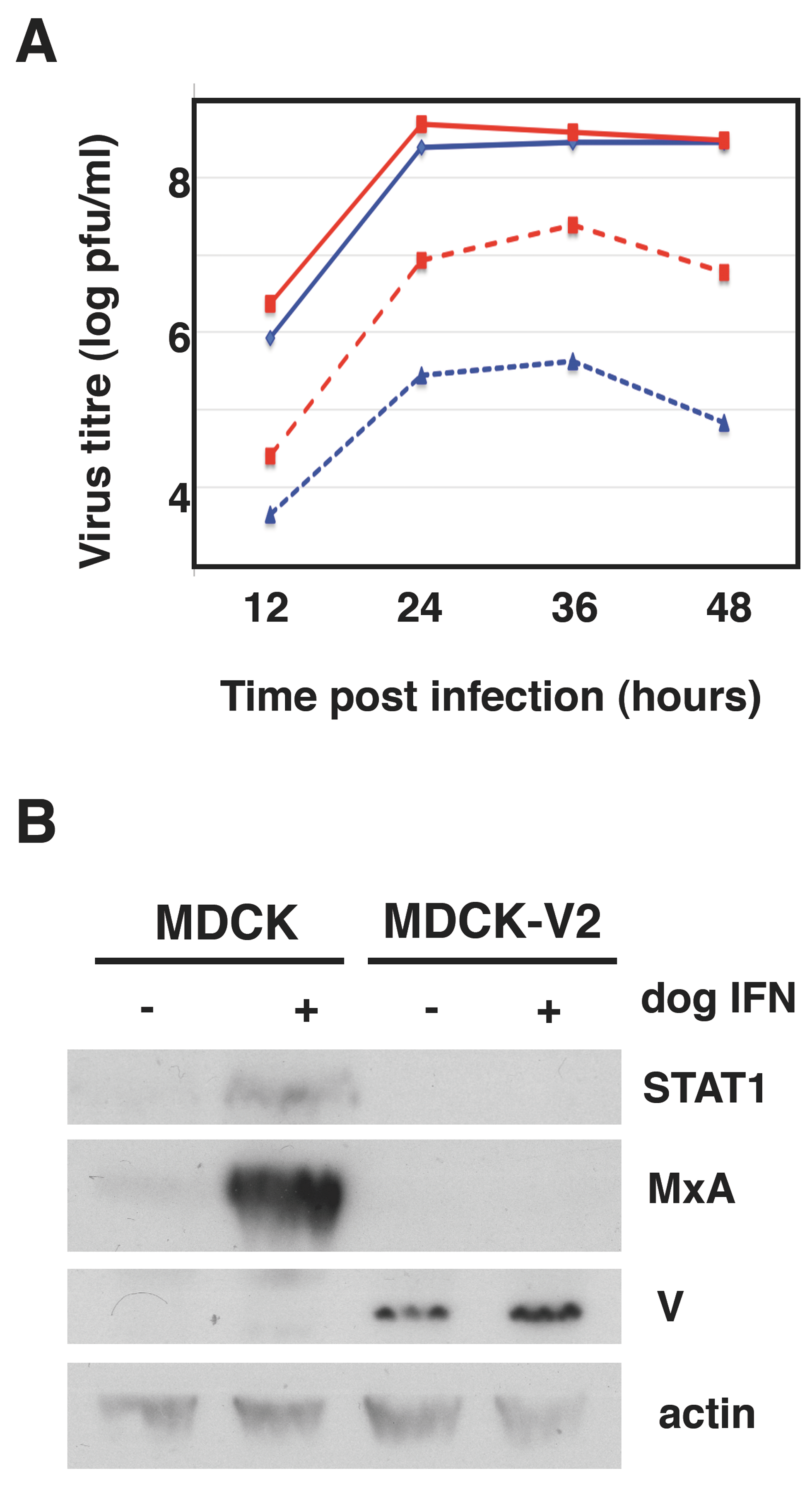

Supplement: Figure S1 — Characterization of MDCK-V2 cells. (A) Cultures of either MDCK-V2 (red lines) of MDCK (blue lines) cells were infected with wt (full line) or DNS1 (dotted line) virus at 0.001 pfu/cell. At the times indicated, samples of the supernatants were withdrawn and virus titres were determined in MDCK-V2 cells. (B) Extracts from MDCK or MDCK-V2 cells were treated or not with dog IFN and analysed by Western blot using anti-STAT1, anti-MxA, anti-V2 or anti-actin antibodies, as indicated. (TIF) [file pone.0098668.s001.tif]

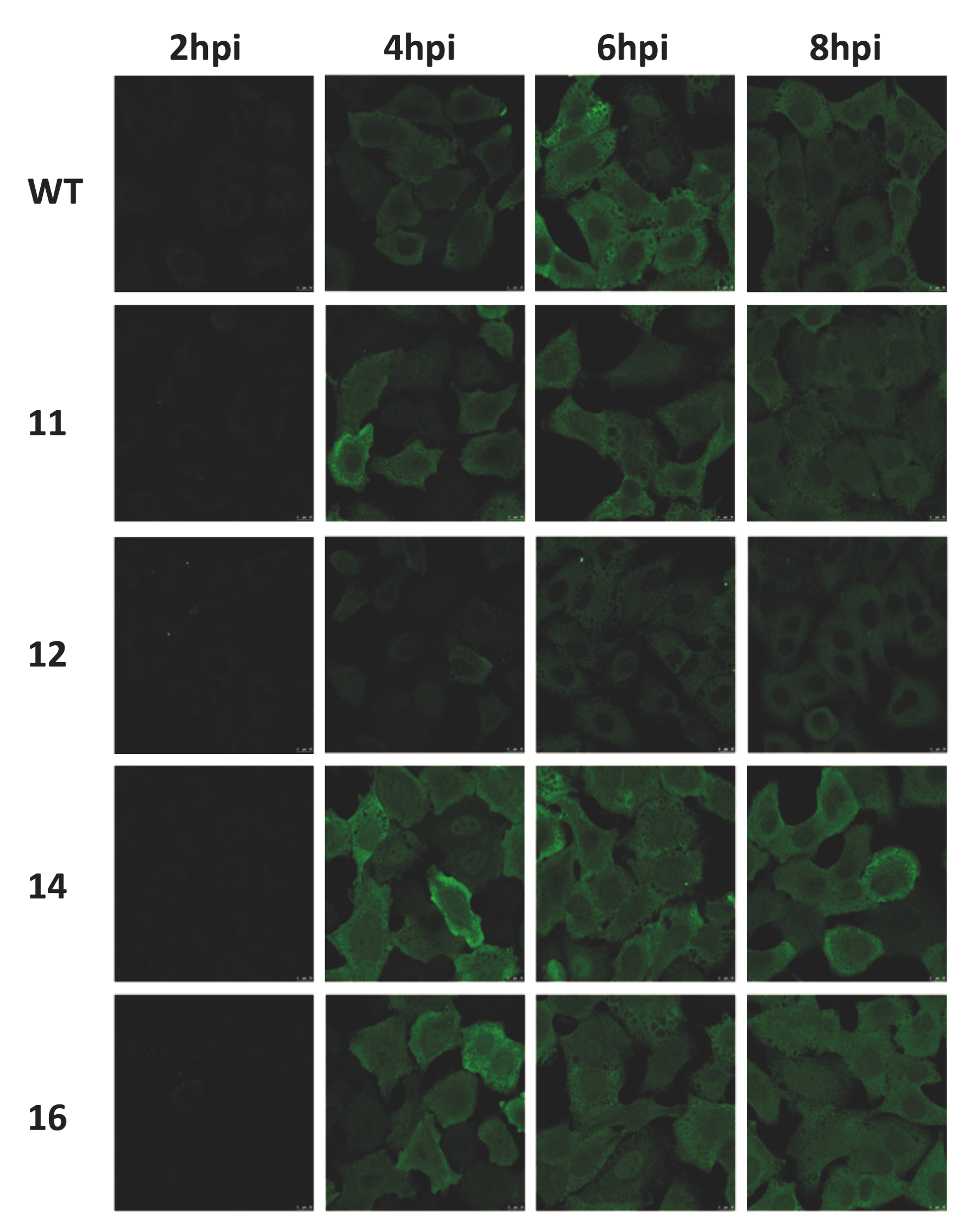

Supplement: Figure S2 — Intracellular localization of NS1 in wt- or mutant virus-infected cells. Cultures of A549 cells were infected with wt or mutant viruses at 5 pfu/cell. At the times indicated the cultures were fixed and processed for immunofluorescence using antibodies specific for NS1. The figure shows projections of representative fields. (TIF) [file pone.0098668.s002.tif]
